# American Institutions for the Insane
*1. Of the Maine Insane Hospital, at Augusta, for 1853.2. Of the Massachusetts Lunatic Hospital, at Worcester, for 1853.3. Of the New York Insane Asylum, at Utica, for 1853.4. Of the Bloomingdale Asylum, New York City, for 1853.5. Of the New Jersey Lunatic Hospital, at Trenton, for 1853.6. Of the Pennsylvania Hospital for the Insane, near Philadelphia, for 1853.7. Of the Pennsylvania State Hospital, at Harrisburg, for 1853.8. Of the Western Asylum of Virginia, at Staunton, for 1853.9. Of the Ohio State Asylum, at Columbus, for 1852 and 1853.


**Published:** 1855-07-01

**Authors:** 


### AMERICAN INSTITUTIONS FOR THE INSANE.*

We are again indebted to Dr. Pliny Earle for the subjoined analysis, published in the last number of our able contemporary, the "American Journal of Medical Science," of the reports of the principal American Asylums for the Insane. Commencing with the *Maine Asylum*, it appears that the number of

| | Men. | Women. | Total. |
|---|---|---|---|
| Patients at the beginning of the year was . | 50 | 34 | 84 |
| Admitted in course of the year . . . . | 65 | 59 | 124 |
| Whole number . . . . . . . | 115 | 93 | 208 |
| Discharged, including deaths . . . . | 54 | 35 | 89 |
| Remaining at the end of the year . . | 61 | 58 | 119 |
| Of those discharged there were cured . . | 28 | 17 | 45 |
| Died . . . . . . . . . | 11 | 4 | 15 |

Deaths from apoplexy, 4; consumption, 3; general paralysis, 6; pneumonia, 1; and laryngitis, 1.

No epidemic prevailed, and no suicide occurred during the year.

We commend to the physicians of other asylums the method pursued by Dr. Harlow, in reporting the complete list of patients. The following is a synopsis of it.

| | | Discharged cured. | Improved. | Unimproved. | Died. |
|---|---|---|---|---|---|
| Persons admitted | 1033 | 404 | 190 | 234 | 118 |
| „ re-admitted | 194 | 79 | 43 | 33 | 18 |
| „ admitted a 3rd time | 50 | 26 | 2 | 7 | 2 |
| „ „ 4th „ | 17 | 10 | 1 | 3 | 2 |
| „ „ 5th „ | 11 | 8 | 2 | 0 | 0 |
| „ „ 6th „ | 5 | 4 | 1 | 0 | 0 |
| „ „ 7th „ | 5 | 2 | 2 | 0 | 0 |
| „ „ 8th „ | 2 | 1 | 0 | 0 | 1 |
| „ „ 9th „ | 1 | 0 | 0 | 1 | 0 |
| Cases aggregate | 1318 | 534 | 241 | 278 | 141 |

---

they have not only had the most perfect knowledge and recollection of all the relations they stood in towards others, and of the acts and circumstances of their lives, but have in general been remarkable for subtlety and acuteness."

Erskine knew perfectly well, as matter of fact, that persons, truly and fully answering to the requirement of law—namely, being "wholly deprived of understanding and memory"—not knowing what they are doing any more than infants, than brutes, or wild beasts," scarcely ever appear in any court whatever. He says, indeed, "these cases are not only extremely rare, but never can become the subjects of judicial difficulty. In other cases, reason is not driven from her seat, but distraction sits down upon it along with her, holds her trembling upon it, and frightens her from her propriety," an admirable and strikingly characteristic portrait of the malady, at least in one of its forms. (See more in "State Trials," vol. xxvii., Hadfield's Case.) I shall again and again have to trace the same features without dread of rebuke.

* 1. Of the Maine Insane Hospital, at Augusta, for 1853.
2. Of the Massachusetts Lunatic Hospital, at Worcester, for 1853.
3. Of the New York Insane Asylum, at Utica, for 1853.
4. Of the Bloomingdale Asylum, New York City, for 1853.
5. Of the New Jersey Lunatic Hospital, at Trenton, for 1853.
6. Of the Pennsylvania Hospital for the Insane, near Philadelphia, for 1853.
7. Of the Pennsylvania State Hospital, at Harrisburg, for 1853.
8. Of the Western Asylum of Virginia, at Staunton, for 1853.
9. Of the Ohio State Asylum, at Columbus, for 1852 and 1853.

Of these, 71 were homicidal, and 129 suicidal; yet no accident has ever occurred from either of the former, and but two of the latter have destroyed themselves at the asylum. One of the homicidal men, removed from the institution against the advice of its officers, killed a man with an axe, in September, 1853.

Another wing for female patients is to be added to the establishment.

2. The *Massachusetts State Lunatic Hospital* went into operation in 1833. Under the energetic executive guidance of the late Dr. S. B. Woodward, it soon became very extensively known; and it has generally been esteemed, even to a comparatively recent period, not only as one of the best conducted, but also as one of the best constructed establishments of the kind in the country. But its Trustees now assert, in their report prefixed to that of Dr. Chandler, that it "has not only ceased to be regarded as a model institution, but it has fallen into the rear-rank in the march of improvement." The halls are "low-studded, being only eight and a half and nine feet high. They are warmed by furnaces, which are very dangerous, and now nearly worn out. Their ventilation is so imperfect as not to deserve the name. The frequent occurrence of erysipelas is but one of the indices" of these defects. "There are forty-eight strong-rooms, or cells, nearly all of them constructed of solid masonry, with iron doors." Some of them "are totally unfit for human habitations. There is an entire want of suitable yards (airing courts) connected with the buildings."

For these reasons, the Trustees, after having visited twenty-six public institutions, ten of them for the insane, in several of the States, recommend the construction of another establishment, and the evacuation and sale of that which now exists.

Let us follow these gentlemen in their tour of observation. "The Superintendents of the Lunatic Hospitals laid us under particular obligations for their generous courtesy, and the very frank and unreserved manner in which they exhibited their establishments, together with their methods of management and modes of treatment, pointing out improvements and criticizing defects. * * * No cell was found without a wooden floor, a wooden door, and plastered or ceiled walls. There is not a brick and mortar cell, with iron doors, in either of the public establishments of the great States of New York and Pennsylvania, nor in the New Jersey State Lunatic Hospital, the design of which is quite generally regarded as a model.

"In the large establishment on Blackwell's Island, containing at the time of our visit, 574 patients, but two were found locked up, and those only temporarily, for a part of the day. The Superintendent, Dr. Rawney, stated that half-a-dozen strong rooms would be sufficient for that establishment, as not more than five or six a day were ever locked up, and those only for a few hours at a time. These rooms were used as sleeping apartments, and did not differ materially, in size, form, and appearance, from the other rooms occupied by patients, except the doors, which were stronger. These remarks in reference to the strong rooms are applicable to all the other establishments visited by us. In nine hospitals out of New England, containing about 2250 patients, the number found under restraint, by being locked up, was only six. In one instance only had that restraint been continued more than two days, and that one had not exceeded a week.

"In the State Hospital, at Utica, but one person was found under restraint, and that one was confined in what they called a chicken-coop bedstead. There, they were in the process of demolishing their strong rooms, as useless. Their hospital was built some ten years after ours, and their strong rooms were comfortable apartments compared with ours, having wooden floors and plank doors, and were each furnished with a bedstead and bed.

"There are no more perfectly warmed and ventilated establishments in the country than the New York Hospital, the Pennsylvania Hospital, the New Jersey State Lunatic Hospital, the State Lunatic Hospital at Harrisburg, and the State Lunatic Hospital at Utica. In all these establishments, steam is the agent employed, and it gives perfect satisfaction. The plan at Utica, being the most recent, is believed to combine more of modern improvements than any other. The Hospital at Utica, in all its appointments, may justly be regarded as a model institution, although its ground plan, in our opinion, is inferior to that of Trenton and Harrisburg."

That the buildings of the Massachusetts Hospital are very defective as compared with those of the institutions of recent origin, there can be no doubt; but it is very evident that the Trustees have placed those defects in as bold relief as possible, by exhibiting them in the light of the broadest contrasts.

In reference to the practical application of the doctrines of Pinel, the Trustees make the following remark:—

"*Dr. Tuke, being the Superintendent, and possessing the whole control, found but little difficulty in testing them in practice at the York Retreat.*" No "Dr. Tuke," and no man named Tuke, was ever Superintendent of that Institution. We never heard, in England, of *any* Doctor Tuke.* This is not the first time, however, that a *myth* of that name has been introduced into the profession, by persons on this side of the Atlantic. Samuel Tuke, for many years one of the Trustees of the Retreat, and well known by his publications upon insanity, was a tea-merchant, in York. Of the occupation of his ancestor, who was a member of the first Board of Trustees, we know nothing.

We now come to the report of Dr. Chandler.

| | Men. | Women. | Total. |
|---|---|---|---|
| Patients in the Hospital, Dec. 1, 1852 . . | 264 | 268 | 532 |
| Admitted in the course of the fiscal year . | 136 | 152 | 288 |
| Whole number . . . . . . . | 400 | 420 | 820 |
| Discharged, including deaths. . . . . | 134 | 166 | 300 |
| Remaining November 30, 1853 . . . . | 266 | 254 | 520 |
| Of those discharged, there were cured . . | 65 | 80 | 145 |
| Died . . . . . . . . . . | 21 | 20 | 41 |

No patients were received excepting such as were sent by order of the Courts, or by the Overseers of the Poor of towns. The Hospital was intended for a number of patients not exceeding 400; yet, at one time in the course of the year, there were 567. Of the 520 at the close of the year, 216 were foreigners; that is, not natives of Massachusetts; and of the latter number, 140 were Irish. "The Irish," says the report, "are almost invariably State paupers. Only three instances have come to my knowledge of their bills, or any part of their bills, having been paid by themselves, or by their friends."

| | Men. | Women. | Total. |
|---|---|---|---|
| Whole number of patients from 1833 to 1853 inclusive . . . . . . . | 2239 | 2225 | 4464 |
| Discharged, recovered . . . . . | 994 | 1059 | 2053 |
| Died . . . . . . . . . . | 253 | 239 | 492† |

*Causes of Death.*—"Marasmus, 78; consumption, 58; apoplexy and palsy, 53; maniacal exhaustion, 49; epilepsy, 45; disease of heart, 20; disease of brain, 20; suicide 19; lung fever, 18; diarrhœa, 18; erysipelas, 15; old age,

* Dr. Pliny Earle is not aware that there is a Dr. Tuke residing near London.

† In one table the report gives but 491. The number of admissions in 1835, is stated in one place as 113, in another, 119. This discrepancy affects the whole number, making it but 4458, if the 113 be correct. We have quoted from the table in which the number of each sex is given.

13; typhus fever, 11; dysenteric fever, 9; inflammation of the bowels, 8; dropsy, 8; hæmorrhage, 6; gastric fever, 5; cholera, 4; cholera morbus, 4; chronic dysentery, 4; mortification of the limbs, 3; disease of the brain from intemperance, 3; bronchitis, 3; hydrothorax, 3; congestive fever, 2; convulsions, 2; land scurvy, 1; concussion of brain, 1; disease of the bladder, 1; fright, 1; rupture, 1; asthma, 1; cancer, 1; pleurisy, 1; jaundice, 1; chorea, 1."

Dr. Chandler gives a table, in which 406 of the patients who died are included, showing the relationship of insanity to longevity. Its substance is as follows:—

| | Years. | Months. | Days. |
|---|---|---|---|
| Average age of 201 males when attacked . . . | 42 | 8 | 10 |
| Average age of 205 females when attacked . . | 39 | 1 | 10 |
| Average age of the whole number, 406 . . . . | 40 | 11 | 1 |
| Average duration of insanity before admission, 201 males . . . . . . . . . | 4 | 2 | 9 |
| Average duration of life after admission, 201 males . | 1 | 9 | 24 |
| Average duration of life after the attack, 201 males . | 6 | 0 | 3 |
| Average duration of insanity before admission, 205 females . . . . . . . . . | 3 | 3 | 6 |
| Average duration of life after admission, 205 females | 1 | 7 | 29 |
| Average duration of life after the attack, 205 females | 4 | 11 | 5 |
| Average duration of life after attack of the whole number, 406 . . . . . . . . | 5 | 5 | 20 |
| Average age at death, 201 males . . . . | 48 | 8 | 13 |
| Average age at death, 205 females . . . . | 44 | 0 | 15 |

"The chance of life," says the report, "for persons in health at corresponding periods, as calculated and acted upon by life-insurance companies, is four times greater than is here exhibited for the male, and more than five times greater for the female. This shows pretty conclusively that insanity, when not recovered from, tends to shorten life."

3. The movement of patients at the *New York State Asylum*, in course of the fiscal year ending November 30, 1853, was as follows:—

| | Men. | Women. | Total. |
|---|---|---|---|
| Patients at the commencement . . . . | 215 | 210 | 425 |
| Admitted . . . . . . . . . | 251 | 173 | 424 |
| Whole number . . . . . . . . | 466 | 383 | 849 |
| Discharged, including deaths . . . . . | 227 | 176 | 403 |
| Remaining at end of the year. . . . . | 239 | 207 | 446 |
| Of those discharged, there were recovered . | 95 | 74 | 169 |
| Died . . . . . . . . . . . | 19 | 20 | 39 |

Deaths from phthisis, 11; exhaustion, 7; general paralysis, 5; epilepsy, 3; exhaustive mania, 2; apoplexy, cerebral effusion, tumour of brain, pericarditis, hypertrophy and dilatatation of heart, typhoid fever, dysentery, chronic diarrhœa, phlegmonous erysipelas, erysipelas from wounds received prior to admission, contusions received before admission, 1 each.

"The seven cases reported as having died of exhaustion, were wasted by disease or vicious habits. Three of them, at the development of mania, had been purged, blistered, and profusely bled, and were brought to the Asylum on beds. This injudicious treatment cannot be too strongly condemned. The recuperative powers in these were so far exhausted, that no amount of care, stimulation, and nutrition could arouse them. It may be proper to state that a number of persons received in a state of extreme feebleness, after long nursing, watchfulness, and free stimulation, recovered."

"No case of suicide has occurred for more than two years."

The number of men admitted was greater than in any preceding year; that of women was diminished by an inability to receive them, on account of the extensive alterations in their department, necessary for the introduction of the new apparatus for heating and forced ventilation. Sixty applications were rejected, and forty-seven incurable cases discharged, to make room for curables.

In eleven of the cases admitted, the insanity was complicated with epilepsy. "In six of them, epilepsy came on at puberty, preceded the derangement, and was the exciting cause. In others, the epilepsy commenced in childhood, and early induced dementia. In the table of causes, two of these cases are put down to the intemperance of the father, the father being represented as in a state of beastly drunkenness most of the time for a few years previous to their birth. Nine of the whole number of epileptics had a drunken parentage, and, in some, this vice extended several generations back."

Of seven cases in which the mental disorder was accompanied by general paralysis, six were of intemperate parentage, and three of the six "had a drunken and licentious ancestry." In fourteen patients, ten men and four women, the insanity is ascribed to "spiritual rappings."

"No epidemic prevailed during the year. One case of variolous disease appeared in May, which was immediately isolated, and the entire population of the house vaccinated, after which no other case occurred. This was a case of acute dementia, of eight months' standing and seven months' residence. *The mental affection entirely disappeared simultaneously with the full eruption of the disease.*"

There may be imprudent haste, as well as unwise delay, in regard to the removal of insane persons to the institutions devoted to their treatment. Especially is this true when the removal involves a long and wearisome journey, which the patient, disabled by physical disease or debility, is unqualified to bear. A woman mentioned in this report, while labouring under acute puerperal mania, "was brought from a distant State, *a journey of eight hundred miles*, THREE WEEKS AFTER DELIVERY, and was wasted to a skeleton, and not able to speak when received. She had not taken food or drink for four days, nor slept for seventy-two hours." If any reader should have a doubt as to the result of this case, he may remove that doubt by referring to the report.

In the record of general results already quoted, it will be perceived that 169 cases are reported as "recovered." In a subsequent and more specific table, these recoveries are arranged under two heads,—viz., "Well," and "in usual health." Of the former there are 126, and of the latter 42. Our former notices of the reports from Utica have given Dr. Benedict's reasons for this distinction. A similar method of reporting is adopted in some of the German institutions. It is probably a more accurate method than that which is the most generally in vogue.

As the results of the industry of the female patients and attendants, it is stated that they repaired all the clothing and bedding, and made 5760 garments and articles of domestic use. The tailors' shop produced 60 coats, 149 vests, and 205 pantaloons; and the carpenter's shop, numerous articles of household furniture.

| | |
|---|---|
| Aggregate of patients, from Jan. 16, 1843, to Dec. 1, 1853 . | 3923 |
| Discharged, recovered . . . . . . . . . . | 1625 |
| Died . . . . . . . . . . . . . . . . | 446 |

4. From the general statistics of the *Bloomingdale Asylum*, we abstract the following:—

| | Men. | Women. | Total. |
|---|---|---|---|
| Number of patients, Jan. 1, 1853 | 52 | 67 | 119 |
| Admitted in course of the year | 73 | 62 | 135 |
| Whole number " " | 125 | 129 | 254 |
| Discharged, including deaths | 69 | 61 | 130 |
| Remaining, Dec. 31 | 56 | 68 | 124 |
| Of those discharged, there were cured | 21 | 28 | 49 |
| Died | 13 | 9 | 22 |

Deaths from typho-mania, 4; chronic mania, 4; abscess in the brain, 3; epilepsy, 3; *paralysis générale*, 3; puerperal mania, 2; mania-à-potu, 1; apoplexy, 1; serous effusion within the cranium, 1; pleurisy, 1.

Seven of the patients died within one week after admission.

The subjoined extract presents the views of Dr. Brown in regard to the numerical method as applied to insanity:—

"The terms *recovered*, *improved*, and *not improved*, as used in reports of this character, must necessarily be in some degree indefinite in their signification; they represent only the *opinion of the reporter* on the cases embraced in the opposite numerals. That this opinion will be determined, or modified by the observer's temperament, is a fact so well known to those familiar with the subject that the 'statistics of insanity' are very generally regarded as collections of individual opinions, rather than as reliable scientific data. With the sincerest desire to arrive at entire accuracy, it is not unfrequently difficult to determine the exact state of the mind at the moment of the patient's discharge. While, in one instance, we may be discomfited by the sudden relapse and return of one dismissed as convalescent, our chagrin may be smoothed by the assurance that another, whose removal we had strongly resisted as imprudent and critical, has progressed to complete restoration. It may even be somewhat questionable whether that degree of improvement which justifies enrolment among the 'recovered,' can, in every case, be adequately determined as the patient is leaving the asylum, as yet unsubjected to the test of association with the world, and unexposed to influences which may have produced his disease.

"The marked contrariety of opinion as to the justice of characterizing certain phases of a still existing malady as an *improvement*, may well qualify confidence in the numerical method of estimating results of treatment in mental diseases. The subsidence of agitation, noisy declamation, and violence, followed by a prolonged period of calm, does not necessarily indicate a better condition of mind; nor do improved physical health, and discontinuance of bad habits, invariably point toward recovery. Yet, each of these supposed changes is desirable as an improvement on its antecedent state, and while some physicians *exclude* from the class of *improved* all cases in which an approach towards recovery from the mental derangement be not apparent, others with equal respect for truth, *admit* all in which the above-named desiderata are attained."

So fully have we concurred in the opinions advanced in the paragraph last quoted, that, for several years, in making our extracts from the statistics of the reports, we have entirely omitted those under the heads, "much improved," and "improved." That the temperaments of the superintending physicians of the various asylums differ, it is reasonable to suppose; and that the judgment of each physician is somewhat influenced by his specific temperament, is a proposition which will not be contested by any person much versed in physiology and psychology. But, that this influence is sufficient to destroy our confidence in the statistics of *cures*, reported agreeably to a sincere conviction of truth, we cannot believe. If it be, the sooner the practice of reporting them is discontinued, the better will it be for the progress of true science.

There is much truth, as well as appropriateness, in the following remarks near the close of the report before us:—

"We have been too prone to regard the *balance sheet*, the *farm account*, and the *report of articles manufactured* as matter of special solicitude, contemplating the patient as an agent in the industrial hive, rather than as the object of all the accumulated means of treatment. In the lunatic hospital, as in society and in the State, the individual must be prominent. The very disease for which he is admitted tends ultimately to destroy individuality. For this reason his identity must be preserved, his just claims recognised, his self-respect encouraged, and his mind incited to useful or refining occupation. In this kind of moral treatment, some of our co-labourers of the Old World excel us. To emulate their merit, we need a courageous zeal which shrinks from no obstacle, a generous enthusiasm that waits not to weigh restored minds against a diminished credit balance, and the stimulating conviction that laurels yet ungathered line the steeps above us."

Large additions to the "lodges" of the Asylum have recently been erected, and the new method of heating, in connexion with a forced ventilation, introduced into those buildings. The number of applicants for admission into the institution is greater than the means of accommodation. In 1836, there were upwards of 160 patients. The departments now occupied by patients are at least fifty per cent. more extensive than at that time; yet Dr. Brown proposes to limit the number, in future, to 150. This recognition of the importance of sufficient room is one among many evidences of improvement.

| | Men. | Women. | Total. |
|---|---|---|---|
| 5. By the report of Dr. Buttolph, it appears that the number of patients in the *Asylum at Trenton*, Jan. 1, 1853, was | 91 | 91 | 182 |
| Admitted in course of the year | 56 | 63 | 119 |
| Whole number | 147 | 154 | 301 |
| Discharged, including deaths | 49 | 47 | 96 |
| Remaining January 1, 1855 | 98 | 107 | 205 |
| Of those discharged, there were cured | 27 | 26 | 53 |
| Died | 10 | 7 | 17 |

Deaths from general exhaustion, 4; consumption, 3; epilepsy, 3; apoplexy, 4; congestion of brain, 1; congestion of lungs, 1; chronic diarrhœa, 1.

The patients enjoyed "a remarkable exemption from all acute and epidemic diseases" throughout the year.

The liberal donation from Mr. Randolph, mentioned in the last preceding report, has been devoted to the construction of an octagonal stone building, thirty-two feet in diameter, lighted from the top, and surrounded by a portico eight feet in width. The interior will be finished in a style appropriate for a handsome reading-room and museum.

| | Men. | Women. | Total. |
|---|---|---|---|
| Patients admitted from May 15, 1848, to Dec. 31, 1853 | 320 | 314 | 634 |
| Discharged, recovered | 108 | 106 | 214 |
| Died | 36 | 35 | 71 |

To this brief report are appended the "Propositions relative to the construction of Hospitals," and those "On the organization of Hospitals," which have been issued by the Association of Medical Superintendents of American Institutions for the Insane. Had that Association achieved no other good, the production of these two documents would alone have been a sufficient recompence for all its labours. In future, should there be a hospital for the insane erected and put in operation, with the imperfections of those which were established twenty years ago, it will not be for the want of available means for their prevention.

6. The report of Dr. Kirkbride, for 1853, furnishes the following statistics of the movement of the inmates of the *Pennsylvania Asylum for the Insane* in the course of the year :—

| | Men. | Women. | Total. |
|---|---|---|---|
| Patients, December 31, 1852 | | | 215 |
| Admitted since that time | | | 191 |
| Whole number | 205 | 201 | 406 |
| Discharged, including deaths | 93 | 78 | 171 |
| Remaining, Dec. 31, 1853 | 112 | 123 | 235 |
| Of those discharged, there were cured | | | 88 |
| Died | 10 | 5 | 15 |

Deaths from acute mania, 4; softening of the brain, 3; exhaustion from long-continued refusal of food, 2; tubercular consumption, chronic inflammation of the lungs, chronic diarrhœa, disease of the bladder, sloughing of the perineum, and old age, 1 each.

Of seven patients prematurely removed from the hospital, five were believed to be curable.

| | Men. | Women. | Total. |
|---|---|---|---|
| Patients admitted since the opening of the hospital | 1299 | 1099 | 2398 |
| Single | 708 | 427 | 1135 |
| Married | 536 | 530 | 1066 |
| Widowed | 55 | 142 | 197 |
| Cured | 622 | 515 | 1137 |
| Died | 142 | 103 | 245 |

Insanity commenced before the patient was 10 years of age, in 5; between 10 and 20 years, in 282; 20 and 30 years, in 915; 30 and 40, in 577; 40 and 50, in 290; after the fiftieth year, in 229.

During the whole of the past year, "the institution has been rather more than comfortably filled, the average number being 229, while 220 is regarded as the capacity of the building." The highest number was 248. Some applications for admission were refused. The elaborate system of moral management heretofore pursued at this hospital, and pretty fully described in our previous notices of the reports emanating from it, is still continued. We still await, however, the introduction here, as well as at all the other similar establishments in the United States, of one feature in the general treatment, without which it is believed that no institution for the insane can be perfect. We allude to an active, thorough, energetic system of disciplinary, gymnastic, hygienic, physical and mental improvative and curative management of the chronic cases—even of those who may have been more or less demented, torpid, and stupid, and perhaps given up as incurable, for years. In short, a school for idiots, technically speaking, is needed in every large institution for the insane. We have wonderful results from those schools in Germany, Switzerland, France, England, and, to some extent, in this country, where the subjects were congenitally imbecile. We anticipate success no less eminent among those whose demention is acquired, for we are fully convinced that the physical lesion to be overcome is, in a large proportion of cases, a less discouraging obstacle in the latter than in the former. All things, and especially the *power*, by his facilities for the acquisition of the means, point out Dr. Kirkbride as the man to become the pioneer in this undertaking. The hospital under his superintendence already approximates so nearly to perfection, that there is some danger of his becoming the Alexander of his sphere, and weeping that there are no more realms to conquer. But while among his patients, one imbruted remnant of that which was once a man, moves only in obedience to the calls of nature, and of his attendant, perhaps to the latter alone, everything is not accomplished. While along the benches, or on the floors, in corners or partially secluded nooks, lying, sitting, crouching, or standing in listless inactivity, are those who still bear some relic, how slight soever it may be, of their former intellectual manhood, so long will the necessity be indicated for that systematic physical and mental schooling, which has been mentioned. Be it understood that we are pointing to an entirely new era in the history of our institutions specially devoted to the insane; an era within the first gleams of the aurora of which we have been brought by the progress of the last half-century. We believe, that at the present time, the class of patients in question receive no more efficient treatment in any of our Asylums, than in the Pennsylvania Hospital; and we have alluded to them there by partial description, only because we know that such a class exists, and, but too often, a very numerous class, in every establishment of the kind.

Among the important improvements of the past year, mentioned in the report before us, is a serpentine carriage-road through the pleasure-grounds of the department for females. A similar road is in progress through the grounds devoted to the men. When the latter is completed, a drive of one mile and three-quarters can be taken within the walled inclosure of the Hospital.

Gas has been introduced, for lighting the buildings, and, with three times the amount of light formerly furnished by oil, the actual expense is less.

Dr. Kirkbride devotes several pages to the discussion of the question whether insanity be increasing in a greater ratio that the population. "It is not difficult," he remarks, "to understand that there may be elements in operation in this country more likely to produce mental derangement than in most others; but at the same time, it must also be conceded that other causes, elsewhere prevalent, are here absent; and which, different as they are in their general character, tend to produce nearly the same effects. While the general prevalence of comfort among our own population, the comparative ease with which nearly every individual may earn a livelihood, and the absence of tyranny and a grinding oppression of the poor and dependent, ought to contribute no less to the mental than to the physical well-being of the whole community; still, some of the characteristic traits of our people, originating in this happy state of things, tend to a different result. The very active and wide-spread commercial speculations of our citizens, the incessant taxing of the mental and physical powers to their utmost, the absorbing pursuit of business, aiming at rapid success and the hasty accumulation of wealth, is a state of constant mental anxiety, of labour without relaxation; and it is too often a mere lottery, in which great and sudden good fortune is the exception, and loss and disappointment the more common, though less noted results." The man of business, when able to retire, is unfitted for the change; "he discovers, with surprise, that long habit has rendered the excitement, the toil, and the anxieties of business, great as they may have been, among the necessaries of his existence; and irksome as he may occasionally have found them, he now concludes that they are infinitely preferable to the *ennui* which presses so heavily upon him. Fortunate is the man thus situated, who can take a hearty, permanent interest in other pursuits, who can engage in works of benevolence or of public utility that will render him not only a benefactor to his species, but will also preserve him from an indulgence in habits that may ruin him physically, and from yielding to feelings which may seriously impair the functions of the mind." After mentioning other causes, the conclusion is arrived at, that, "it will probably be found that the number of cases (of insanity) among us has *not* increased in a greater ratio than that of the general population." The greater prominence, during the last few years of the subject of insanity, and of *its* subjects, is mentioned as only an apparent, not a real indication of the increase of the disease. The filling up of the hospitals, also, "does not prove that insanity increases more rapidly than the population." "Philadelphia, in 1830, had accommodation for 385 insane, with a population of 188,961. At the end of 1840, with a population of 258,037, she could provide for about 530, and now, with half a million of inhabitants, her different institutions can receive 630 patients." Thus, in regard to that city, the provisions for the cure of the insane have not kept pace with the population.

But further accommodation is needed, and hence Dr. Kirkbride suggests, "that a new Hospital, replete with every modern discovery, and all the improvements suggested by a large experience, and capable of accommodating 200 male patients, should be erected on the seventy acres of land now comprising the farm of this institution, and directly west of its present inclosed pleasure-grounds; while the present buildings, with everything included within our external wall, should be given up for the exclusive use of a similar number of females."

7. We glean from the report of Dr. Curwen, the subjoined sketch of the movement of the population of the *Pennsylvania State Hospital*, in 1853.

| | Men. | Women. | Total. |
|---|---|---|---|
| Patients in the Hospital, Dec. 31, 1852 | 59 | 47 | 106 |
| Admitted in course of the year | 95 | 68 | 163 |
| Whole number | 154 | 115 | 269 |
| Discharged, including deaths | 55 | 32 | 87 |
| Remaining, December 31, 1853 | 99 | 83 | 182 |
| Of those discharged, there were cured | | | 27 |
| Died | | | 17 |

*Causes of Death.*—Epilepsy, 5; exhaustion consequent to chronic mania, 5; paralysis, 3; "disease of the lungs," 2; acute inflammation of the brain, 1; gradual decay of the vital powers, 1.

"The general health of the household has been good. We have been spared the visitation of any epidemic, and only a few cases of disease incident to the season were under treatment during the summer and autumn." "Several of those who were much improved at the time of their removal, subsequently regained their former mental vigour." "A little girl, three years and four months old, evincing unequivocal symptoms of mental disorder, was admitted in the early part of the year. The mental disorder was recent. This case, so interesting on account of the age and mental peculiarities, still continues under treatment."

In the table of supposed causes we find the following: "Millerism, 1; spiritual rappings, 1; religious excitement, 2."

Small libraries have been established in some of the wards. "Pictures of a cheerful character hung on the walls, and mottoes suggestive of pleasant ideas, and printed in large letters, have been introduced into the wards, more particularly of the excited classes." The donations from Philadelphia, collected by Miss Dix, and mentioned in our notice of the report for 1852, amounted to $5182. "The museum and reading-room buildings have been finished. They are 42 feet long, by 25 feet wide. A portico runs nearly the whole length of the front, from which a very pleasant view is obtained. They are placed one on either side of the front of the building; and each is easily accessible from the wards of the sex for which it is intended. It is proposed, so far as can be done, to procure the mineral and geological productions of the different parts of the Commonwealth, and to give to each county so much room as may be needed to exhibit the specimens obtained."

8. In the twelve months preceding the 30th September, 1853, the number of patients at the *Western Asylum of Virginia*, exceeded, by twenty-two, that of any preceding year. No malignant or epidemic disease occurred among them; neither was there a case of suicide. Of the 1264 patients received since the opening of the Asylum, only five have terminated their existence with their own hands. In two of these there was no certainty that the death was not accidental.

| | Men. | Women. | Total. |
|---|---|---|---|
| Patients at the beginning of the year . . . | 202 | 138 | 340 |
| Admitted in course of the year . . . . | 69 | 51 | 120 |
| Whole number . . . . . . . . . | 271 | 189 | 460 |
| Discharged, including deaths . . . . . | 54 | 29 | 83 |
| Remaining at the end of the year . . . | 217 | 160 | 377 |
| Of those discharged, there were cured . . | 26 | 15 | 41 |
| Died . . . . . . . . . . . | 17 | 10 | 27 |

The diseases terminating fatally are not reported. Among the causes of insanity, we observe that the "excessive use of tobacco" is mentioned in *three* of the cases, "inhaling tobacco fumes" in *one*, and the "excessive use of tobacco and ardent spirits" in *one*.

Of the 460 cases, the insanity *commenced* before the age of 20 years, in 67; between 20 and 30, in 160; between 30 and 40, in 93; between 40 and 50, in 50; after the fiftieth year, 18; unascertained, 72. The great preponderance of the decennium from 20 to 30 years will be perceived.

| | Men. | Women. | Total. |
|---|---|---|---|
| Aggregate of patients admitted since July 1, 1836 | 711 | 474 | 1185 |
| Discharged, cured . . . . . . . . | 270 | 184 | 454 |
| Died . . . . . . . . . . . | 150 | 86 | 236 |

Aside from the statistical tables, the report of Dr. Stribling is almost exclusively occupied in the description of improvements recently made upon the premises, and the suggestion of others. Gas was introduced for the purpose of lighting the apartments, on the 1st of January, 1853. "We are now satisfied," says the report, "that the institution can be supplied from the gas-works with an amount of light far greater than that which it formerly derived from oil, lard, and candles, *for a small fraction of what these materials cost.*"

As this establishment and the Eastern Asylum can accommodate but about 700 patients, and as there are within the State, according to the last census, 922 insane whites, and 945 white idiots, many of the latter probably not congenitally idiotic, the Doctor urges upon the Legislature "to make at once a liberal appropriation for the erection of another Asylum" for 250 patients.

9. On the 1st of July, 1852, Dr. S. Hanbury Smith retired from the superintendence of the *Ohio Lunatic Asylum*, and was succeeded by Dr. Elijah Kendrick. The reports heretofore emanating from this institution have been more voluminous than those from any other similar establishment in the country, with perhaps a single exception. The one now before us is of more restricted limits.

| | Men. | Women. | Total. |
|---|---|---|---|
| Patients in the Asylum Nov. 15, 1851 . . | 150 | 151 | 301 |
| Admitted in course of the fiscal year . . | 149 | 126 | 275 |
| Whole number " " " . . . . | 298 | 277 | 576 |
| Discharged, including deaths . . . . | 169 | 147 | 316 |
| Remaining, Nov. 15, 1852 . . . . . | 130 | 130 | 260 |
| Of those discharged, there were cured . . | 70 | 71 | 141 |
| Died . . . . . . . . . . | 37 | 21 | 58 |

Deaths from consumption, 13; diarrhœa, 8; dysentery, 8; epilepsy, 4; marasmus, 4; maniacal exhaustion, 4; typhoid fever, 4; gastritis, 2; anæmia, 2; inanition, 2; suicide, 2; apoplexy, organic lesion of brain, caries of vertebræ, typhoid pneumonia, and erysipelas, 1 each.

Of the cases admitted, the mental derangement of 22 is ascribed to "religious anxiety," and that of 26 to "spirit rappings." In the latter class, Dr. Kendrick remarks, that "the suicidal tendency is especially prominent, while the constant resting of the thoughts upon the scenes of an imaginary world renders it more difficult to attract attention to those of the real. Such cases, though recent, have proved more unfavourable than many others of the same class."

*Thirty-eight* of the patients received had previously been inmates of the Asylum, and discharged *recovered*. *Sixteen* of them had been absent less than a year. When will the physicians of all our institutions for the insane report this item of their statistics?—an item of more importance than many which they regularly place before us.

Of the 275 persons admitted, the insanity commenced before the age of 20 years in 45; between 20 and 30 years, 94; 30 and 40 years, 65; 40 and 50, 41; 50 and 60, 23; 60 and 70, 6; 70 and 80, 1.

From the fact that one hundred and fifty-one applications for admission were rejected in the course of the year, we infer that Ohio is beginning sorely to feel the want of another hospital.

The bodies of deceased patients not reclaimed by their friends, have heretofore been privately interred. The funerals are now conducted openly, and in the presence of many of the patients. The circumstances which induced this change, and the results of the experiment, are thus related:—

"On a visiting tour through the grounds, my ear caught the following dialogue between two patients at work. Said A.: 'What disposition do you suppose is made of our bodies after death here?' B. replied: 'In my opinion, the doctors boil us up.' 'Very true,' continued A., 'that may be the fate of some; but my opinion is, that many of us are taken to doctors' shops, so have our bones picked and stuck up to view as our bodies are here.' From that moment I was resolved, if possible, to dispel this mental delusion. Accordingly, on the first occurrence of a death, the chaplain, the officers, and assistants, accompanied by many of the male patients, followed the deceased to his final resting-place. Here they were addressed by the chaplain, in language chaste and appropriate, in every way calculated to convince their understanding that not only were they fed, clothed, and cared for during their lives, but that, at their deaths, they should not be forgotten. The effect was strikingly impressive.

"In this first experiment we realized our highest hopes. Many who were denied the privilege, reproached us for not having permitted them to unite with their friends in rendering the last tribute of respect to a departed fellow-sufferer. We still observe all the rites and ceremonies due and proper on such occasions; take out at all times a large number of patients, both male and female, and nothing indecorous or disorderly has yet transpired to interrupt the practice. So far as we are capable of judging, the influence has been salutary and controlling."

Dr. Kendrick does not give a very flattering description of the condition of the buildings in regard to the facilities for promoting the comfort and restoration of the inmates. The water-closets and bathing apparatus, "in plain terms, are a disgrace to the institution." There are no means of forced ventilation, and the patients' "sleeping apartments are not warmed." He recommends an appropriation of $33,800 to remedy these and other defects.

Report for 1853:—

| | Men. | Women. | Total. |
|---|---|---|---|
| Patients remaining Nov. 15, 1852 | 130 | 130 | 260 |
| Admitted in course of the fiscal year | 110 | 129 | 239 |
| Whole number „ „ „ | 240 | 259 | 499 |
| Discharged, including deaths | 125 | 122 | 247 |
| Remaining Nov. 15, 1853 | 115 | 137 | 252 |
| Of those discharged, there were cured | 71 | 62 | 133 |
| Died | 12 | 12 | 24 |

*Causes of Death.*—Phthisis pulmonalis, 7; inanition, 4; maniacal exhaustion, 3; typhus fever, 2; bilious remittent fever, 1; congestive fever, 1; variola, 1; pleuropneumonia, 1; paralysis, 1; ulceration of bowels, 1; exhaustion from journey, 1; suicide, 1.

A case of smallpox was "developed, under most inexplicable circumstances, in the male department," and every precaution was taken to prevent the propagation of the disease among the patients. No other case occurred until two months afterwards, when a female patient was attacked, had the disease mildly, and recovered. An endemic fever, of a mixed character, commenced among the inmates about the middle of July, attained its height about the middle of August, and continued, "sporadically," to the time at which the report was written. "Though commencing as a common bilious remittent, owing to the hospital tendency, after the first few days it assumed the typhoid or typhous type; and again, in the case of convalescents, at the end of two weeks, reassumed the remittent form. Among the patients there were 31 cases." These were mostly of males, and one of them ended fatally. There was, also, one case of congestive fever, of which the patient died. Of the 31, "through the renovating influences of physical disease, and the necessary remedial agents used for their recovery, 13 were restored to reason concurrently with their convalescence from the fever. Several were also much improved mentally, but again relapsed. In all the cases, even in the demented, the mind seemed more clear during the attack than when in usual physical health."

Sixteen cases of the fever occurred among the employées of the institution, one of them terminating in death. Dr. Kendrick attributed the disease to malaria rising from the "illy constructed sewers and most offensive cesspools," and from the earth thrown up in digging numerous ditches for steam and water-pipes through "the sub-soils charged with the accumulated impurities of years." The disease was treated, in its early stage, with alteratives, aperients, and diaphoretics; when typhoid symptoms arose, by the addition of tonics and stimulants, and, upon the reassumption of the remittent type, by anti-periodics and tonics.

Of the 239 patients admitted, 68 had suffered from former attacks of insanity. Among the "probable causes" of the disease, "religious excitement" ranks the highest in numbers, 32 being assigned to it. It is evident that the etiology of the disease is differently viewed by different physicians. Dr. Stokes, of the Mt. Hope Institution, asserts, in one of his late reports, that he has never seen a case clearly traceable to the cause in question. Eleven cases are attributed to "spirit rappings." "For some of these," says Dr. K., "my sympathies have been strongly awakened, and, though deprecating the impious folly, I cannot refrain from here entering my feeble protest against the *indiscriminate* commitment of such persons to lunatic asylums." He then quotes some medico-legal remarks of such tenor as to lead the reader to the inference that, in some of the patients alluded to, there was no delusion or insanity, other than that which might exist in regard to the so-called "spiritual manifestations."

Forty-eight of the patients admitted had the suicidal propensity. Thirty-five of them had attempted self-destruction. Of the thirty-five, thirteen had recovered from their mental disease at the close of the year.

| | Men. | Women. | Total. |
|---|---|---|---|
| Whole number of patients, 1839 to 1853, inclusive | 1220 | 1135 | 2355 |
| Discharged recovered | 601 | 570 | 1171 |
| Died | 188 | 135 | 323 |

*Diseases which proved Fatal.*—"Exhaustion and general decay, without discoverable local lesion, 56; consumption, 52; dysentery, 31; diarrhœa, 30; epilepsy, 28; fever, 28; inanition, 26; apoplexy, 16; palsy, 9; inflammation of the lungs, 7; dropsy, 6; inflammation of the brain, 4; inflammation of the liver, 3; chronic inflammation of the peritoneum, 3; tabes mesenterica, 3; suicide, 3; inflammation of the pericardium, 2; inflammation of the stomach, 2; erysipelas, 2; organic lesion of the brain, 1; caries of the vertebræ, 1; chronic inflammation of the bronchia, 1; bilious colic, 1; ulceration of the bowels, 1; inflammation of the kidney, 1; cancer of the womb, 1; cutaneous cancer, 1; gangrene of the face, 1; exhaustion from journey, 1; confluent smallpox, 1; cause not assigned, 1."

The State Legislature has made appropriations for warming the buildings by steam, in connexion with forced ventilation, and for the construction of an infirmary.

---

